# Creating new business, economic growth and regional prosperity through microbiome‐based products in the agriculture industry

**DOI:** 10.1111/1751-7915.12698

**Published:** 2017-02-27

**Authors:** Brajesh K. Singh

**Affiliations:** ^1^ Hawkesbury Institute for the EnvironmentWestern Sydney UniversityPenrith2751NSWAustralia; ^2^ Global Centre for Land‐Based InnovationWestern Sydney UniversityPenrith2751NSWAustralia

The manipulation of plant and environmental microbiomes is becoming an increasingly popular tool to sustainably increase farm productivity for food and nutrient security (Singh and Trivedi, 2017). There are multiple drivers for the increasing focus on microbiome tools in agriculture, including the need for a substantial increase in food production from shrinking arable lands. For example, an increase of 70% in farm productivity is needed by 2050 to meet demand of the continually growing global population. Continuous land degradation means that farm productivity increases need to be met from 8 to 20% less arable land. In addition, climate change, the increasing frequency of extreme weather events, the high cost and unreliable supply of phosphorus fertilizers, combined with the structural decline in soil fertility means better approaches to utilize available natural resources, are considered a priority. Harnessing the plant and soil microbiome is increasingly being recognized as one such approach. Environmental (e.g. minimizing water and air pollution) and social (e.g. consumer's demand of chemical‐free food and environment preservation) pressures are other key drivers that provide the impetus towards green and renewable technologies for sustainable farming.

## Initial outcomes from microbiome approaches and emerging industries

Although still in its infancy, the use of and market for, microbiome‐derived tools (the term used here is broad in nature and include both the whole microbial community as well as single species products) in agriculture is growing rapidly. The current market for agricultural biologicals (including plant extracts) is ~$2.9 billion. This is dwarfed by the agrochemical market of ~$240 billion which comprises both fertilizers and pesticides. However, biopesticides account for over $2 billion annually which constitutes ~5% of $44 billion annual market of chemical pesticides (Reed and Green, 2013). Within the biopesticide market, microbially derived products account for more than 50% of the biopesticide market. Going forward, it is projected that microbiome‐based products will have a comparable market size to chemical agrochemicals within the next few years. For example, by 2020, there will be more biopesticides in the European market than chemical pesticides. It is likely that this trend will continue throughout the globe as consumers increasingly demand high nutritional value food produced with no/minimal chemical inputs. Consumer demand is also driven by the growing links between a clean environment and improved public health outcomes.

There are a number of microbial products in the market that provide measurable benefits to crop productivity and quality. Further, there are several other products in the pipeline/nearing market launch by well‐established companies (e.g. Bayer, Monsanto) and emerging companies (e.g. Indigo, Novozymes). For example, Indigo is launching a new cotton seed product coated with microbes which is reported to provide ~10% yield benefits when cotton is grown under water stress. Similarly, Bioag Alliance (a joint venture between Monsanto and Novozyme) has announced a number of new microbial products which can substantially increase corn productivity by enhancing nutrient uptake and nematicidal activities. Increasing investment by both government agencies (e.g. USA Microbiome Initiative; International Bioeconomy Forum) and private sectors, combined with a large number of start‐up companies suggest that microbiome products for agriculture use will exponentially increase in the near future, propelled by sophisticated technologies (e.g. next‐generation sequencing, nanotechnology, synthetic biology) for analysing and manipulating environmental microbiomes. Such technologies fuel the optimism for new and effective biopesticides by advancing scientific knowledge of plant and soil microbiomes. Indeed, the vast majority of biopesticides are still to be discovered. For example, approximately 60% of human drugs are derived from natural resources (predominantly from microbes and plants; Singh and Macdonald, 2010) as a result of intensive research and investment in the medical field. This compares to only 11% of pesticides originating from natural resources. A renewed focus on microbiomes can result in the discovery of more effective biopesticides and biofertilizers (Timmis *et al*., 2014). Exploration of new microbial strains and products for agricultural use is greatly helped by the increasing number of complete microbial genomes on public and private databases.

Current microbial products typically contain one or several microbial species and represent microbes that can be cultured. However, culturable microbes constitute only 1% of the total microbial population, and thus, there is huge potential residing in the untapped microbes. In the future, real benefits could be obtained from our ability to engineer solutions using the entire microbiome *in situ*. Our ability to manipulate entire microbiomes *in situ* could open up an entirely new industry which could include ‘green’ chemistry, microbial cocktails and/or based on synthetic biology. In addition, microbes provide crucial genes for genetically modified (GM) plants (also called biotech crops), the fastest adopted new technology in modern agriculture (James, 2015), despite legal and public perception hurdles. The current annual market for biotech crops is estimated to be over $15 billion. The genes that provide either pest control and/or herbicide resistance are generally obtained from microbes and transferred into plants. One of the real success stories is GM cotton farming containing insecticidal genes from *Bacillus thuringiensis*. Microbial genes are also used to increase the nutritional quality of food products. For example, a gene from *Pantoea ananatis*, in combination with a plant gene, was used to create ‘Golden Rice’ to increase the concentration of vitamin A in rice grains to meet the dietary requirements of large populations in developing countries. All approaches described above will benefit from the ability to tap into the ever‐increasing metagenomic data produced from microbiome research and allow the identification of additional genes and microbial products, without the need for culturing, to provide agricultural yield benefits. These beneficial microbial products/genes can be used in future to manipulate the microbiome *in situ*, or used to genetically modify crops for more efficient production.

## Role in regional prosperity, job creation and boosting activities of allied industries

The wider use of microbiome tools in agriculture can significantly increase regional growth if the demand and supply routes are regionally locked. For example, microbial agents or products for crop productivity could be isolated, produced in local facilities and supplied locally rather than purchased from different regions and countries (Reed and Green, 2013). These facilities will provide jobs while farmers will have access to microbial agents which are native to local regions and, therefore, potentially have better efficacy. This can bring significant social prosperity along with economic growth to local facilities that produce the microbial agents and products. Similar approaches have been successfully trialled in some Asian and South American countries.

**Figure 1:**
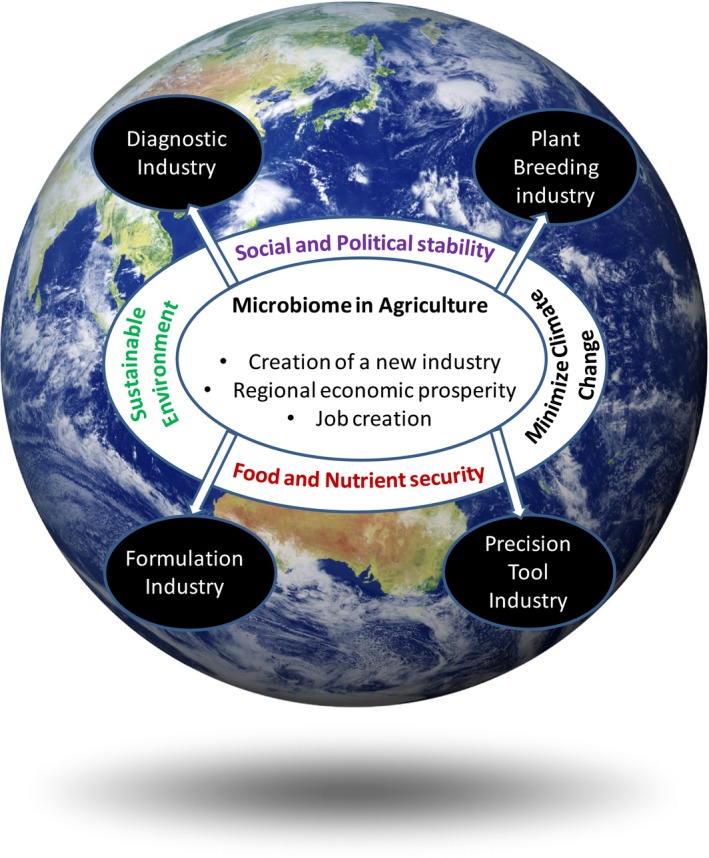
Microbiome tools and products in agriculture offer an unprecedented opportunity for creation of new industries and long‐term global economic growth. Sustained investment will result in critical outputs including creation of new industries comparable to the agrochemical industry, regional prosperity via local production and use. These outputs will directly contribute towards food and nutrient security and a sustainable environment by reducing chemical use, minimizing impact on environment and non‐target organisms, moderating the rate of climate change by reducing greenhouse gas emission and promoting social and political stability by providing new jobs and economic growth at local and national levels. Microbiome tools in agriculture will also boost innovation and commercialization of allied industries such as the diagnostic and testing industry, plant breeding, formulation and precision tools industries. Above activities together can make a significant contribution towards long‐term global economic growth and environmental sustainability.

Additionally, microbiome approaches will boost the activities of other allied sectors such as diagnostics and testing, formulation and plant breeding industries which all can contribute to the national and global economic growth and will result in better environmental and social outcomes. For example, the global market for Agriculture and Environmental Diagnostics is estimated to be valued at over $7.7 billion by 2020 (MCP‐1091 report) driven mainly by the globalization of food supply, policy requirements and consumer demands. Over 90% of this diagnostic market is covered by testing for *Salmonella* and *Listeria* spp in food products. This market is currently dominated by the 3M Company and Biomerieux SA, with a number of new start‐up companies recently entering the arena. In the near future, consumer demands will accelerate testing for chemical residues, GMOs and the origin of food products. Additionally, microbial products will require different formulations and delivery technologies requiring new or modified technologies. Similarly, a new approach is being considered for the plant breeding industry where the retention and promotion of the plant beneficial microbiome are being considered. These requirements will promote innovations and commercialization to boost productivity of the allied industries and contribute significantly to economic growth, regional prosperity and job creation (Fig. 1).

## Challenges and outlooks

All indicators suggest the use of microbiome tools and microbial products in agriculture have great potential to create new industries and boost economic growth and job creation. This is already evident by the significant investments (billions of dollars) made by both government agencies and private sectors. However, to realize the vast and real potential of the microbiome, in addition to overcoming scientific challenges (Singh and Trivedi, 2017), microbiome tools and products need to meet crucial industrial standards. These include: (i) meeting the gold standard of efficacy and microbial products need to work in most environmental and climatic conditions and provide efficacy comparable, if not better, to agrochemicals. (ii) Be economically and logistically competitive in terms of price, transportation and storage. (iii) Avoid regulatory hurdles by actively contributing towards global regulation mechanisms and by self‐monitoring. (iv) Be socially responsible by sharing knowledge and benefits with all stakeholders (industry, farmers, environmentalists and the general public). Such an approach should avoid negative public perceptions such as those previously experienced by the biotech crop industries. Making efficacy and environmental impact assessment data open access, along with a regulatory framework with explicit recognition of societal benefits, would help to secure and maintain public support (Singh, 2010). The societal benefits could be demonstrated via sourcing regional products, creating regional jobs (e.g. fermentation, formulation and application of local products) and demonstrating environmental, nutritional and health benefits (chemical‐free food and clean environment). If industries and regulatory agencies are able to address the above challenges, microbiome approaches in agriculture and agribusiness are poised to offer unprecedented opportunities for sustainable food and nutrient security, creating new and boosting allied industries which will significantly contribute to the national and global economic growth and boost regional prosperity with better environmental and social outcomes.
